# The Inhibitory Effects of Ficin on *Streptococcus mutans* Biofilm Formation

**DOI:** 10.1155/2021/6692328

**Published:** 2021-03-23

**Authors:** Yan Sun, Wentao Jiang, Mingzheng Zhang, Lingjun Zhang, Yan Shen, Shengbin Huang, Mingyun Li, Wei Qiu, Yihuai Pan, Liang Zhou, Keke Zhang

**Affiliations:** ^1^Department of Endodontics, School and Hospital of Stomatology, Wenzhou Medical University, Wenzhou 325027, China; ^2^School and Hospital of Stomatology, Wenzhou Medical University, Wenzhou 325027, China; ^3^Institute of Stomatology, School and Hospital of Stomatology, Wenzhou Medical University, Wenzhou 325027, China; ^4^State Key Laboratory of Oral Diseases, National Clinical Research Center for Oral Diseases, West China Hospital of Stomatology, Sichuan University, Chengdu 610041, China; ^5^Department of Stomatology, Nanfang Hospital, Southern Medical University, Guangzhou 510515, China; ^6^Affiliated Cixi Hospital, Wenzhou Medical University, Cixi 315300, China

## Abstract

To investigate the effects of ficin on biofilm formation of conditionally cariogenic *Streptococcus mutans* (*S. mutans*). Biomass and metabolic activity of biofilm were assessed using crystal violet assay, colony-forming unit (CFU) counting, and MTT assay. Extracellular polysaccharide (EPS) synthesis was displayed by SEM imaging, bacteria/EPS staining, and anthrone method while acid production was revealed by lactic acid assay. Growth curve and live/dead bacterial staining were conducted to monitor bacterial growth state in both planktonic and biofilm form. Total protein and extracellular proteins of *S. mutans* biofilm were analyzed by protein/bacterial staining and sodium dodecyl sulfate polyacrylamide gel electrophoresis (SDS-PAGE), severally. qRT-PCR was conducted to detect acid production, acid tolerance, and biofilm formation associated genes. Crystal violet assay, CFU counting, and MTT assay showed that the suppression effect of ficin on *S. mutans* biofilm formation was concentration dependent. 4 mg/mL ficin had significant inhibitory effect on *S. mutans* biofilm formation including biomass, metabolic activity, EPS synthesis, and lactic acid production (*p* < 0.05). The growth curves from 0 mg/mL to 4 mg/mL ficin were aligned with each other. There was no significant difference among different ficin groups in terms of live/dead bacterial staining result (*p* > 0.05). Protein/bacterial staining outcome indicated that ficin inhibit both total protein and biofilm formation during the biofilm development. There were more relatively small molecular weight protein bands in extracellular proteins of 4 mg/mL ficin group when compared with the control. Generally, ficin could inhibit biofilm formation and reduce cariogenic virulence of *S. mutans* effectively in vitro; thus, it could be a potential anticaries agent.

## 1. Introduction

Dental caries, a biofilm relevant disease, is caused by destruction of mineralized tooth tissue due to acidic catabolites from the bacterial fermentation of sugars [1]. The Global Burden of Disease Study 2016 revealed that caries of permanent teeth had the greatest prevalence, decayed permanent, and deciduous teeth ranked in second and fifth place, respectively, in highest incidence diseases [2]. *Streptococcus mutans* (*S. mutans*), a major extracellular polymeric substances producer, is the most well-known biofilm forming bacteria in oral cavity and was reported as most associated microbe in the transition of oral flora from a healthy one to a cariogenic one [3]. It has been proven that decrease or eradication of *S. mutans* can diminish or prevent caries development [4]. The major virulence factors of *S. mutans* include adhesion, acid production, and acid tolerance. Adhesion enables *S. mutans* to colonize in the oral cavity and form biofilm. Extracellular polymeric substances produced by *S. mutans* that is composed of extracellular polysaccharides (EPS), extracellular DNA (eDNA), proteins, and lipoteichoic acids provide crucial scaffold for biofilm and are essential for expression of cariogenic virulence [5]. Biofilm, as a natural barrier, could facilitate adhesion of microbes, promote cooperation and communication within microorganisms, obtain oxygen and nutrients more effectively for microbe, and protect microorganism from external environment [6]. Biofilm showed more resistant to antimicrobial agents than that of planktonic microorganism, which could be up to 1000 times [7].

To solve biofilm-associated problems above, current adopted strategies were mainly through antibiotics or antimicrobial-based ways and physical-mechanical approaches [8]. One of the effective antibiofilm strategies is enzymatic degradation of biofilm, which displays a favorable prospect as it is natural, biodegradable, minimally invasive, and is with great advantage of rare resistance [9]. The mechanisms of enzymatic degradation to control dental biofilm vary with different types of enzymes. Human matrix metalloprotease-1 inhibited and disrupted *Enterococcus faecalis* (*E. faecalis*) biofilm through bacterial growth inhibition and degradation of biofilm matrix proteins [10]. Proteases such as bromelain, actinidin, papain, proteinase K, and trypsin were also reported to inhibit dental biofilm including single species and multispecies biofilm [11, 12]. Other than proteolytic enzymes, some enzymes targeted polysaccharide components of biofilms. Dispersin B could act on extracellular polysaccharide by cleaving polymers of *β* (1, 6) N-acetylglucosamine to suppress biofilm formation or promote biofilm dispersal [13, 14]. Mutanases and dextranase were able to control dental biofilms such as *S. mutans* biofilm effectively by specifically hydrolyzing (1 → 3)-*α*-glucoside links and (1 → 6)-*α*-glucosidic linkages, respectively [15, 16]. A chimeric glucanase comprising mutanase and dextranase was proved to prevent dental biofilm formation availably [17]. Besides, nuclease was another option for controlling dental biofilm. DNase I could inhibit *S. mutans* biofilm formation effectively by digesting eDNA [18, 19]. Currently, a nuclease called DeoC was identified as a *S. mutans* biofilm dispersal factor and it could facilitate *S. mutans* flee from neutrophil extracellular traps [20].

Ficin, a sulfhydryl protease isolated from the latex of fig trees, can cleave proteins at the carboxyl side of methionine, lysine, arginine, glycine, serine, threonine, valine, asparagines, alanine, and tyrosine [21]. Further, it was proved to be a bifunctional enzyme as it had the intrinsic peroxidase-like activity [21]. Recently, researchers found that ficin disrupted *Staphylococcus aureus* (*S. aureus*) and *Staphylococcus epidermidis* (*S. epidermidis*) biofilms and enhanced the antibiofilm effects of antibiotics [22]. Moreover, ficin had no toxic effect on dog adipose derived stem cells and MCF7 carcinoma cells [22]. Ficin is endowed with wider antibiofilm potential as it is a nonspecific sulfhydryl protease and can disrupt the biofilm matrix backbone. So far, there was no report studied the effect of ficin on conditional cariogenic *S. mutans* biofilm. Therefore, we conducted this study to investigate the antibiofilm activity of ficin against *S. mutans* biofilm. Concretely, biomass (by crystal violet assay and CFU counting), metabolic activity (by MTT assay), and cariogenic virulence involving EPS (by SEM imaging, bacteria/EPS staining and anthrone method) and acid production (by lactic acid assay) were to value the suppression effect of ficin on *S. mutans* biofilm. Then, we tested if antibiofilm mechanism of ficin was relevant to bacterial growth inhibition (by growth curve and live/dead bacterial staining) and biofilm extracellular proteins degradation (by protein/bacterial staining and sodium dodecyl sulfate polyacrylamide gel electrophoresis). Finally, expression of acid production, acid tolerance, and biofilm formation-associated genes were monitored by qRT-PCR.

## 2. Materials and Methods

### 2.1. Bacterial Strains and Growth Conditions

*S. mutans* UA159 was obtained from the Institute of Stomatology, Wenzhou Medical University. Brain-heart infusion (BHI, OXOID, Basingstoke, UK) was used for planktonic *S. mutans* growth, while BHIS (BHI supplemented with 1% sucrose, *m*/*v*) was used for biofilm formation. The culture condition was 37°C with 5% CO_2_.

### 2.2. Biofilm Susceptibility Assay

Microdilution method was conducted for detecting the effect of ficin (CAS No. 9001-33-6, MP Biomedicals) on biofilm formation. In brief, the total culture volume was 200 *μ*L, twofold serial dilutions of ficin range from 0 mg/mL to 16 mg/mL were prepared in 96-well plate and overnight culture of *S. mutans* was diluted to final concentration of 10^6^ CFU/mL. 0.12% chlorhexidine (CHX) was used as a positive control group. After incubation for 24 h, the biofilms were tested using crystal violet assay. The biofilms were washed twice with phosphate-buffered saline (PBS) and fixed with methanol for 15 min. Then, the biofilms were stained by 0.1% (*w*/*v*) crystal violet and were quantified by addition of 150 *μ*L 33% acetic acid to each crystal violet-stained well; the absorbance was measured at 590 nm (A590) with a microplate reader (SpectraMax M5, Molecular Devices, USA) [23]. For biofilm qualitative analysis, snapshots of crystal violet-stained biofilms were captured by a stereomicroscope (Nikon SMZ800N, Nikon Corporation, Japan).

The concentration of ficin at 1 mg/mL, 2 mg/mL, 4 mg/mL, and 8 mg/mL was used for following experiments, and 0 mg/mL, 0.12% CHX, protease inhibitor (PI), and PI+4 mg/mL ficin (PI+4) served as control groups. The biofilms were cultured on glass slides in 24-well plates.

### 2.3. Colony-Forming Unit (CFU)

The 24 h biofilms formed on glass slides in 24-well plate were washed twice with PBS, followed by scraping with sterilized blades and sonication/vortexing in PBS [24]. Then, collected bacterial suspensions were gradient diluted and spread onto BHI agar plates to support *S. mutans* growth for 48 h. After that, the CFU were analyzed.

### 2.4. MTT Metabolic Assay

MTT metabolic assay was conducted as described before [25]. The 24 h biofilms on glass slides in 24-well plate were transferred to a new 24-well plate and treated with MTT solution (0.5 mg/mL) for 1 h. Then, the glass slides were put into another 24-well plate and immersed by dimethyl sulfoxide (DMSO) for 20 min in the dark. Subsequently, 200 *μ*L mixed solution was used for absorbance measurement (A540).

### 2.5. Lactic Acid Production

The glass slides with 24 h biofilms in 24-well plate were rinsed with cysteine peptone water (CPW) to detach floating bacteria. Then, they were transferred to a new plate containing buffered peptone water (BPW) supplemented with 0.2% sucrose. The whole system was cultured at 5% CO_2_, 37°C for 3 h, and BPW was used to equilibrate the pH of the system to ensure acid generation. The lactic acid production was measured by lactate dehydrogenase (LDH) with a standard curve method [26].

### 2.6. Scanning Electron Microscopy (SEM)

SEM was used to observe the structure of *S. mutans* biofilms. For biofilm formation, *S. mutans* (1 × 10^6^ CFU/mL) in BHIS supplemented with 0 mg/mL, 1 mg/mL, 2 mg/mL, 4 mg/mL, and 8 mg/mL of ficin, 0.12% CHX, protease inhibitor, both protease inhibitor, and 4 mg/mL ficin, severally. After 24 h, biofilms formed on glass slides in 24-well plate were rinsed and fixed with 2.5% glutaraldehyde for 12 h. Then, the specimens underwent dehydration of gradient ethanol and examined by scanning electron microscope (SEM, Quanta 200, FEI, Hillsboro, OR, USA) at magnifications of 5,000x [27].

### 2.7. Extracellular Polysaccharides Assay

The bacteria/extracellular polysaccharides (EPS) were stained by fluorescein to observe the architecture [27]. At the beginning of culturing biofilms, 2.5 *μ*M Alexa Fluor 647-dextran conjugate (Molecular Probes, Invitrogen Corp., Carlsbad, CA, USA) was supplemented into the culture media. After 24 h, the biofilms formed on glass slides in 24-well plate were stained with 2.5 *μ*M SYTO 9 (Molecular Probes, Invitrogen Corp., Carlsbad, CA, USA) for 30 min. The bacteria were stained green by SYTO 9 (excitation/emission channel are 480 nm/500 nm) and the polysaccharides were stained red by Alexa Fluor 647-dextran conjugate (excitation/emission channel are 650 nm/668 nm). The stained images were captured by confocal laser scanning microscope (CLSM, Nikon A1, Nikon Corporation, Japan), a 60x objective lens. The ratio between EPS and bacteria was calculated according to the coverage using Image pro plus 6.0 (Media Cybernetics, Inc., Silver Spring, MD, USA) [28].

Anthrone method was used to quantify the water-insoluble exopolysaccharides [29]. Briefly, the 24 h biofilms formed on glass slides in 24-well plate were collected and resuspended in 0.4 mol/mL NaOH. After centrifugation, the supernatant reacted with anthrone reagent at 95°C for 6 min, followed by reading the optical density (OD) at 625 nm.

### 2.8. Live/Dead Bacterial Viability Assay

The BacLight live/dead bacterial viability kit (Molecular Probes, Eugene, OR, USA) was used to detect bacterial viability. The 24 h biofilms formed on glass slides in 24-well plate were stained with 2.5 *μ*M SYTO 9 and 2.5 *μ*M propidium iodide for 30 min severally, following manufacturer's instructions. Live bacteria were stained green (excitation/emission channel are 480 nm/500 nm), and dead bacteria were stained red (excitation/emission channel are 490 nm/635 nm) using CLSM (Nikon A1, Nikon Corporation, Japan). Each sample was obtained at five randomly selected views with a 60x objective lens. The ratio between live and total bacteria was analyzed on the basis of coverage with software Image pro plus 6.0 (Media Cybernetics, Inc., Silver Spring, MD, USA) as mentioned above.

### 2.9. Protein and Bacterial Staining

Protein and bacterial staining was conducted according to previous study [30]. We monitored the changes in protein at a series of time points (2 h, 4 h, 8 h, 16 h, and 24 h) during the biofilm development in 24-well plate. 20 *μ*M SYTO 63 (Molecular Probes, Eugene, OR, USA) was added to specimen for 30 min to stain bacteria (excitation/emission channel are 657 nm/673 nm); subsequently, the specimen was incubated with 100 *μ*g/mL FITC (excitation/emission channel are 495 nm/525 nm) for 1 h to stain proteins. The bacteria were stained red while proteins were stained green. The biofilms formed on glass slides were examined by CLSM (Nikon A1, Nikon Corporation, Japan) with a 60x objective lens.

### 2.10. Extracellular Proteins Isolation and SDS-Polyacrylamide Gel Electrophoresis (PAGE) Analysis

The biofilm extracellular proteins were isolated as previously reported [31]. Briefly, the biofilm formed on glass slides in 24-well plate was handled with 0.1 mol/L NaOH with 1 mM EDTA and incubated at 0°C for 1 h. After centrifugation at 3000 g and 4°C for 30 min, the supernatant was collected, 3 volume of precooled acetone were added and incubated at -20°C overnight. After centrifugation again at 3000 g and 4°C for 30 min, the sediment was used for further SDS-PAGE analysis. 50 *μ*g of extracted proteins were loaded, and 10% separating gel was used for electrophoresis. After stained with Coomassie blue, the protein bands were captured by a camera.

### 2.11. RNA Extraction and Quantitative Real-Time PCR (qRT-PCR)

*S. mutans* was collected from 24 h biofilm formed on glass slides in 24-well plate by centrifugation, and RNA was extracted using a Trizol reagent (Invitrogen, USA) [32]. The RNA concentration and purity were determined by a nanodrop 2000, while the RNA integrity was detected by agarose gel electrophoresis. cDNA was synthesized by a PrimeScript™ RT Master Mix (Perfect Real Time) kit (Takara, Japan). qRT-PCR was analyzed by TB Green™ Premix Ex Taq™ II (Tli RNaseH Plus) kit (Takara, Japan) in a Step One Plus Real-Time PCR System (Applied Biosystems, CA, USA). The volume of reaction mixture was set as 20 *μ*L (10 *μ*L 2× TB Green Premix Ex Taq II, 0.4 *μ*L 50× ROX Reference Dye, 2 *μ*L cDNA, 0.8 *μ*L Forward Primer, 0.8 *μ*L Reverse Primer, 6 *μ*L H_2_O). The reaction conditions were 95°C for 30 s, 40 cycles (95°C for 5 s, 55°C for 30 s, and 72°C for 30 s). The gene expression was normalized with reference gene 16S using 2^−ΔΔCT^ method. Primers were listed in Table 1.

### 2.12. Data Analysis

All the experiments were repeated three times. One-way analysis of variance (ANOVA) was conducted to identify the significant effects of variables, followed by the Tukey's multiple comparison test (*p* value of 0.05) with the SPSS software 16.0 (SPSS Inc., Chicago, IL, USA).

## 3. Results

### 3.1. Ficin Decreased the Biomass, CFU, and Metabolic Activity of *S. mutans* Biofilm

**Figure 1:**
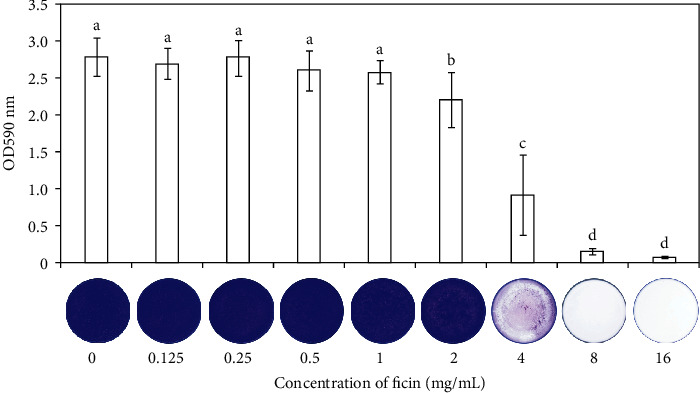
Crystal violet staining of *S. mutans* biofilm. Data are presented as mean ± standard deviation, and values with dissimilar letters are significantly different from each other (*p* < 0.05).

**Figure 2:**
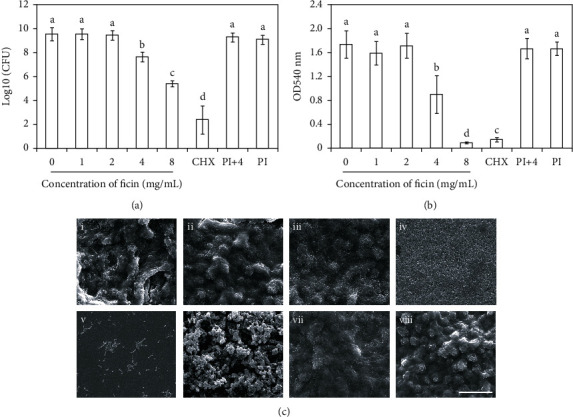
(a) CFU of *S. mutans* biofilm; (b) metabolic activity of *S. mutans* biofilm revealed by MTT assay; (c) SEM images of *S. mutans* biofilm; i-v represent *S. mutans* biofilm under effect of 0, 1, 2, 4, and 8 mg/mL ficin, respectively; vi-viii represent CHX group, PI group, and PI+4 group, severally. CHX represents 0.12% chlorhexidine, PI represents protease inhibitor, and PI+4 represents protease inhibitor+4 mg/mL ficin. Bar = 20 *μ*m. Data are presented as mean ± standard deviation, and values with dissimilar letters are significantly different from each other (*p* < 0.05).

The crystal violet results revealed that suppression effect of ficin on *S. mutans* biofilm formation was dose-dependent, and 4 mg/mL ficin had megascopic inhibitory effect on *S. mutans* biofilm formation (Figure 1). The CFU results showed that there was no significant difference between 0 mg/mL ficin group, 1 mg/mL ficin group, 2 mg/mL ficin group, PI group, and PI+4 group (Figure 2(a), *p* > 0.05). While 4 mg/mL ficin group and 8 mg/mL ficin group reduced by 1.9 log_10_ CFU and 4.1 log_10_ CFU, respectively, when compared with 0 mg/mL ficin group (Figure 2(a), *p* < 0.05). The CHX group showed the least CFU, reaching to 2.4 log_10_ CFU (Figure 2(a)). The biofilm metabolic activity revealed by MTT assay showed similar trend (Figure 2(b)). The OD values in 4 mg/mL ficin group, 8 mg/mL ficin group, and CHX group reduced significantly when compared with 0 mg/mL ficin group (*p* < 0.05).

### 3.2. Ficin Degraded Polysaccharide Production of *S. mutans* Biofilm

Through the SEM images, there was aggregated *S. mutans* being wrapped in numerous extracellular polysaccharides in 0 mg/mL ficin group, 1 mg/mL ficin group, 2 mg/mL ficin group, PI group, and PI+4 group, forming robust biofilms (Figure 2(c)). While for 4 mg/mL and 8 mg/mL ficin group, there were bare extracellular polysaccharides, and biofilms showed had relatively loose structure (Figure 2(c)). In CHX group, there were plenty of swollen and broken bacteria, only few thallus with complete structure in biofilm (Figure 2(c)). The extracellular polysaccharides in this group were also much less than those of 0 mg/mL ficin group, 1 mg/mL ficin group, 2 mg/mL ficin group, PI group, and PI+4 group (Figure 2(c)).

**Figure 3:**
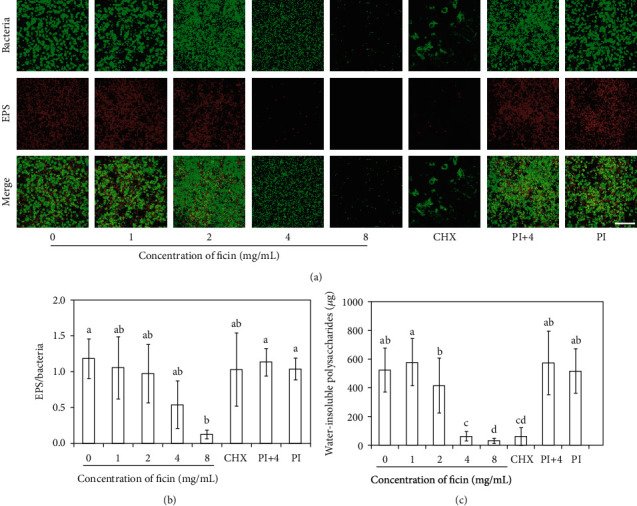
(a) EPS/bacterial staining of *S. mutans* biofilm. Bacteria was stained green, and EPS was stained red; (b) EPS/bacteria ratio according to the EPS staining results; (c) water-insoluble polysaccharides of *S. mutans* biofilm; CHX represents 0.12% chlorhexidine, PI represents protease inhibitor, PI+4 represents protease inhibitor+4 mg/mL ficin. Bar = 50 *μ*m. Data are presented as mean ± standard deviation, and values with dissimilar letters are significantly different from each other (*p* < 0.05).

The bacteria/extracellular polysaccharides staining result presented the same trend as that of water insoluble glucan result (Figure 3). Extracellular polysaccharides stained red and bacteria stained green. The bacteria in 0 mg/mL ficin, 1 mg/mL ficin, 2 mg/mL ficin, PI, and PI+4 groups were wrapped in abundant polysaccharides, while in 4 mg/mL, 8 mg/mL ficin and 0.12% CHX groups showed much less bacteria and polysaccharides (Figure 3(a)). The ratio between polysaccharides and bacteria revealed by quantitative analysis verified there was no significant difference among all the groups except the 8 mg/mL ficin group which had lowest ratio (Figure 3(b)).

The bacterial water insoluble glucan in biofilms were detected by anthrone method. In general, 4 mg/mL ficin group, 8 mg/mL ficin group, and CHX group synthesized remarkably less water insoluble glucan, down to 11.2%, 5.3%, and 11.7% of 0 mg/mL ficin group, respectively (Figure 3(c), *p* < 0.05).

### 3.3. Ficin Reduced Lactic Acid Production of *S. mutans* Biofilm

**Figure 4:**
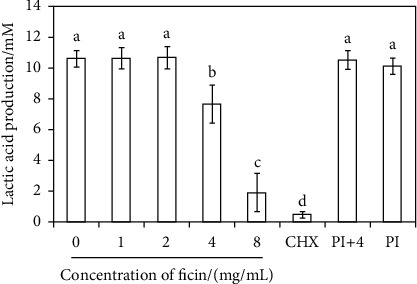
Lactic acid production of *S. mutans* biofilm. CHX represents 0.12% chlorhexidine, PI represents protease inhibitor, and PI+4 represents protease inhibitor+4 mg/mL ficin. Data are presented as mean ± standard deviation, and values with dissimilar letters are significantly different from each other (*p* < 0.05).

The lactic acid production results displayed that 4 mg/mL ficin group, 8 mg/mL ficin group, and CHX group produced less lactic acid than other groups (Figure 4, *p* < 0.05). 4 mg/mL ficin group and 8 mg/mL ficin group reduced lactic acid production to 72.2% and 18.3% of 0 mg/mL ficin group (Figure 4).

### 3.4. Ficin Inhibited *S. mutans* Biofilm Formation Not Mainly through Affecting Bacterial Vitality

**Figure 5:**
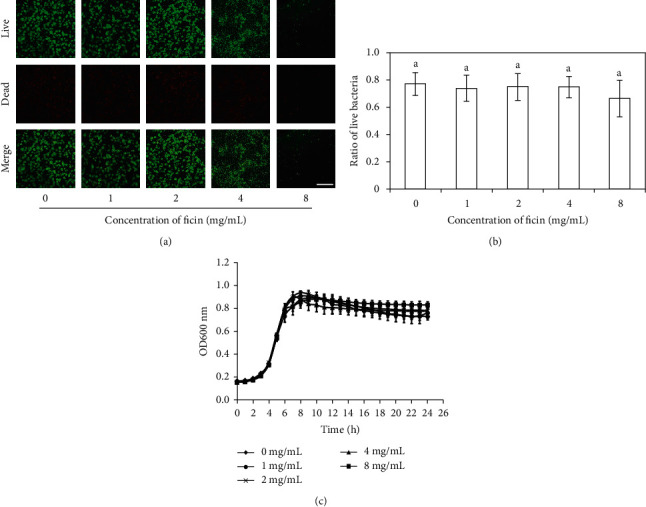
Live/dead bacterial staining of *S. mutans* biofilm. (a) Live bacteria was stained green, and dead bacteria was stained red when merged; (b) ratio of live bacteria according to live/dead bacterial staining results; (c) growth curve of *S. mutans* under different concentration of ficin. Bar = 50 *μ*m. Data are presented as mean ± standard deviation, and values with dissimilar letters are significantly different from each other (*p* < 0.05).

To study whether ficin suppressed *S. mutans* biofilm formation was through influencing bacterial activity, growth curve and live/dead bacterial staining in biofilms were managed (Figure 5). The live/dead bacterial staining in biofilms showed the bacterial vitality seemed uninfluenced by ficin (Figure 5(a)). There was no significant difference between different ficin groups in live/dead bacterial staining result according to quantitative analysis (Figure 5(b), *p* > 0.05). The growth curves of 0 mg/mL ficin group, 1 mg/mL ficin group, 2 mg/mL ficin group, 4 mg/mL ficin group, and 8 mg/mL ficin group were aligned with each other roughly (Figure 5(c)).

### 3.5. Ficin Inhibited *S. mutans* Biofilm Formation and Reduced Protein during the Biofilm Development

**Figure 6:**
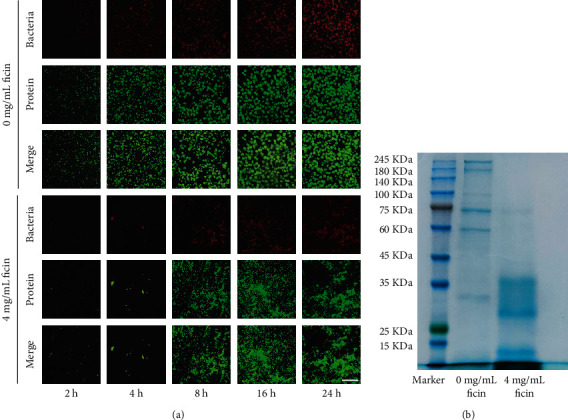
(a) Protein and bacterial staining of *S. mutans* biofilm. Bacteria was stained red and protein was stained green; (b) SDS-PAGE analysis of extracellular proteins of *S. mutans* biofilm (B). Bar = 50 *μ*m.

We stained the total protein during the biofilm development and found 4 mg/mL ficin-reduced bacteria (stained red) and total protein (stained green) in biofilm when compared with 0 mg/mL ficin group in 2 h, 4 h, 8 h, 16 h, and 24 h (Figure 6(a)). From these results, ficin could reduce total protein in biofilm and inhibit biofilm formation and during the biofilm development (Figure 6(a)). Further protein staining results revealed by Coomassie blue staining showed that there were more relatively small molecular weight protein bands in 4 mg/mL ficin group when compared with 0 mg/mL ficin group in extracellular protein (Figure 6(b)).

### 3.6. Ficin Changed Gene Expression of *S. mutans* in Biofilm

**Figure 7:**
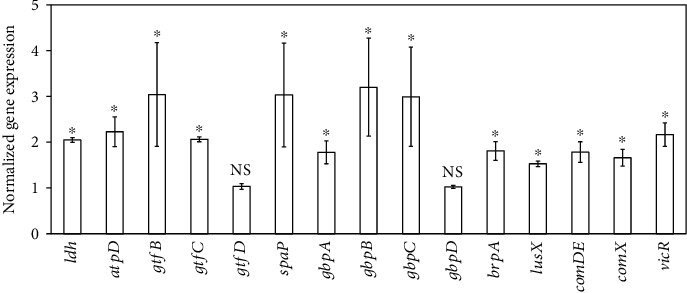
The mRNA expression levels of *ldh*, *atpD*, *gtfB*, *gtfC*, *gtfD*, *brpA*, *gbpA*, *gbpB*, *gbpC*, *gbpD*, *spaP*, *lusX*, *comDE*, *comX*, and *vicR* in *S. mutans* biofilm (^∗^*p* < 0.05; NS, *p* > 0.05).

Compared with 0 mg/mL ficin group, acid production and tolerance-associated *ldh* and *atpD* were upregulated significantly in 4 mg/mL ficin group (Figure 7, *p* < 0.05). Polysaccharide- and biofilm-related genes *gtfB*, *gtfC*, and *gtfD* were about 3.0-fold (*p* < 0.05), 2.1-fold (*p* < 0.05), and 1.0-fold (*p* > 0.05) in 4 mg/mL ficin group, respectively (Figure 7). The expression of other biofilm-associated genes *brpA*, *gbpA*, *gbpB*, *gbpC*, *spaP*, *lusX*, *comDE*, *comX*, and *vicR* in 4 mg/mL ficin group were also upregulated notably (*p* < 0.05) except *gbpD* (Figure 7, *p* > 0.05).

## 4. Discussion

In this study, we investigated the effects of ficin on biofilm formation of *S. mutans*. Our results showed that ficin suppressed the biofilm formation of *S. mutans* as well as acid production and EPS synthesis evidently in a dose-dependent manner. We also found that ficin inhibited biofilm formation during the biofilm development. Besides, the total protein was reduced, and there was more relatively small molecular weight extracellular protein under effect of 4 mg/mL ficin. Ficin seemed not influence bacterial vitality markedly under our test, while most of biofilm-associated genes of *S. mutans* detected were upregulated observably when treated with 4 mg/mL ficin.

Acid production is one of the most important cariogenic virulence factors of *S. mutans*. Lactate dehydrogenase encoded by *ldh* plays an important role in the process of carbohydrate metabolism [34]. We found lactic acid production in 4 mg/mL ficin group decrease by 27.8% while the expression of *ldh* was upregulated by 2 times when compared with 0 mg/mL ficin group. The CFU in 4 mg/mL ficin group was reduced by 1.9 log_10_CFU when compared with 0 mg/mL ficin group. Taken these results together, we speculated that ficin could reduce the total lactic acid production of biofilm, while the reduced total lactic acid production of biofilm in 4 mg/mL ficin group may mainly result from the reduction of biofilm biomass. The expression of *atpD* gene which encoded F0F1-H/F-ATPase *β* subunit of the F1 protein was about 2-fold in 4 mg/mL ficin group when compared to 0 mg/mL ficin group. The F0F1-ATPase facilitated the survival of *S. mutans* under acidic environment through removal of protons from the cell to maintain the cytoplasmic pH which was closely related with acid tolerance [39]. We conjectured that ficin upregulated the expression levels of acid tolerance-linked gene *atpD* might respond to the upregulation of acid production gene *ldh*.

EPS, particularly glucans, make great contribution to cariogenicity of *S. mutans*. Water-insoluble glucans among glucans are identified as major components of EPS in *S. mutans* biofilms [40]. EPS can promote adhesion and accumulation of bacteria, provide mechanical stability for extracellular polymeric substances of biofilm, guard bacteria within biofilm from adverse environment, stockpile energy source, a strict substance spread, condense iron, and nutrients in biofilm [41]. The EPS was decreased prominently when the concentration of ficin were 2 mg/mL or higher. While the expression of EPS-related gene *gtfB* and *gtfC* raised dramatically excepted *gtfD*. Gtfs being able to convert sucrose to glucan have three distinct protein, GtfB (encoded by *gtfB*), GtfC (encoded by *gtfC*), and GtfD (encoded by *gtfD*) produce water insoluble glucan, a mixture of insoluble and soluble glucans, and soluble glucan, severally [42]. It was reported that expression of EPS was necessary for adhesion under stress [43]. The upregulated *gtfB* and *gtfC* might result from protection mechanism of *S. mutans* which increased the expression of EPS-associated gene to response to ficin stress.

Adhesion that can facilitate colonization and biofilm formation is another cariogenicity of *S. mutans*. Apart from *gtfB*, *gtfC*, and *gtfD* mentioned above, we detected adhesion-associated genes under the effect of ficin including *spaP*, *gbpA*, *gbpB*, *gbpC*, and *gbpD*. Most of these genes including *spaP*, *gbpA*, *gbpB*, and *gbpC* were upregulated signally except *gbpD*. SpaP (encoded by *spaP*) is a critical adhesin that mediates initial sucrose independent adherence of *S. mutans* to tooth surface by interacting specifically with salivary agglutinin [44]. Then, the stronger adherence, sucrose-dependent adherence, is mediated by glucosyltransferases (Gtfs) and glucan-binding proteins (Gbps). Gtfs, especially GtfB and GtfC, support early attachment and aggregation of bacteria at the early-biofilm phase. Gbps, which cover GbpA (encoded by *gbpA*), GbpB (encoded by *gbpB*), GbpC (encoded by *gbpC*), and GbpD (encoded by *gbpD*), mediate the binding of bacteria to glucans and biofilm formation of *S. mutans* especially GbpB and GbpC [36]. Besides, the gene expression of *brpA* in ficin containing group was also upregulated significantly. Cell surface-associated biofilm regulatory protein BrpA (encoded by *brpA*) played a key role in environmental stress responses and efficient biofilm formation of *S. mutans* [33].

Complex signal regulation systems are involved in biofilm formation. Quorum sensing (QS) that can sense microbial numbers, environmental stresses, and carbohydrate alteration is vital in initial adherence and biofilm maturation [45]. QS-associated genes *luxS*, *comDE*, and *comX* were all upregulated when administered by ficin. *luxS* is responsible for autoinducer-2 synthesis which is involved in interspecies communication, and its deficiency will weaken biofilm formation [37]. *comDE* and *comX* take part in competence-stimulating peptide- (CSP-) induced signal cascade for development of genetic competence and intraspecies communication. *comDE* encodes competence-stimulating peptide (CSP) receptor (*comD*) and response regulator (*comE*), while *comX*, also known as *sigX*, is a sigma factor and is identified as the master regulator of competence development of *S. mutans* [45]. It has been reported that *comD*-, *comE*-, and *comX*-deficient mutants formed biofilms with decreased biomass and lacked architectural integrity [46]. The expression of *VicR*, a response regulator of *VicRK* two component system that can regulate biofilm formation, was also upregulated under ficin [45]. The upregulation of most genes above which could facilitate biofilm formation or necessary for robust biofilm formation including adhesion and signal regulation systems relevant might result from similar reason as that of EPS-associated genes. Under stress of ficin, *S. mutans* upregulated biofilm-associated genes to promote biofilm formation [39].

Antibiofilm mechanism was dissimilar depending on different type of protease. According to previous report, ficin suppressed staphylococcal biofilm by degradation of the protein backbone of biofilm without bactericidal effect [22]. We tested if the antibiofilm effect of ficin on *S. mutans* was through bacterial activity repression, while our data showed that bacterial activity of both planktonic cells, and bacteria in biofilms were not inhibited markedly which was consistent with previous reports [22]. The total protein and extracellular protein in biofilm were reduced, and there were more small molecular proteins under influence of ficin. Hence, the inhibitory effect of ficin on *S. mutans* biofilm formation might also be via degradation of biofilm matrix proteins. It should be noted that ficin inhibited *S. mutans* biofilm formation throughout the whole process of biofilm development. This might be attributed to the degradation of biofilm formation-related protein or polypeptide by ficin. The actual molecular mechanisms of ficin on *S. mutans* biofilm needed to be further studied.

Chlorhexidine, a commonly used oral cavity cleanser, showed toxic towards cells even at concentration that much lower than clinical use. By comparison, previous study used MCF7 cells and dog adipose-derived stem cells to cytotoxicity of ficin; they found ficin was safe for potential biomedical applications [22]. Exploring cocktail treatment that combined ficin with bactericide which had synergetic effect could be ideal antibiofilm strategy in which ficin was used for antibiofilm while bactericide was used for killing residual biofilm and planktonic bacteria. However, lots of work should be done for further application of ficin. For example, dental plaque is more than complex; other biofilm model such as saliva biofilm or animal experiment in vivo were also recommended for further application of ficin in oral cavity. Moreover, as a nonspecific protease, broader antibiofilm effect of ficin was recommended to explore. What is more, potential application of ficin in material modification or tissue engineering such as modify ficin on the surface of materials or incorporate ficin into scaffold could be further studied. For example, recent research showed both antibiofilm and wound-healing properties of chitosan-immobilized ficin [47].

## 5. Conclusions

Based on the fact that ficin had suppressed biofilm formation and cariogenic virulence of *S. mutans* including acid production and EPS synthesis significantly, ficin may be a potential antibiofilm agent to control dental caries.

## Figures and Tables

**Table 1:** Primers used in this study.

Primers	Nucleotide sequence (5′-3′)	References
*16S*-f	CCTACGGGAGGCAGCAGTAG	[33]
*16S*-r	CAACAGAGCTTTACGATCCGAAA	
*ldh*-f	AAAAACCAGGCGAAACTCGC	[34]
*ldh*-r	CTGAACGCGCATCAACATCA	
*atpD*-f	TGTTGATGGTCTGGGTGAAA	[34]
*atpD*-r	TTTGACGGTCTCCGATAACC	
*gtfB*-f	AGCAATGCAGCCAATCTACAAAT	[33]
*gtfB*-r	ACGAACTTTGCCGTTATTGTCA	
*gtfC*-f	CTCAACCAACCGCCACTGTT	[33]
*gtfC*-r	GGTTTAACGTCAAAATTAGCTGTATTAGC	
*gtfD*-f	ACAGCAGACAGCAGCCAAGA	[33]
*gtfD*-r	ACTGGGTTTGCTGCGTTTG	
*spaP*-f	TCCGCTTATACAGGTCAAGTTG	[35]
*spaP*-r	GAGAAGCTACTGATAGAAGGGC	
*gbpA*-f	TCATCAGGCACAGAACCACC	[36]
*gbpA*-r	CAGTTGAGGCTCGTTTCCCT	
*gbpB*-f	ATGGCGGTTATGGACACGTT	[33]
*gbpB*-r	TTTGGCCACCTTGAACACCT	
*gbpC*-f	TCTGGTTTTTCTGGCGGTGT	[36]
*gbpC*-r	GTCAATGCTGATGGAACGCC	
*gbpD*-f	TTGACTCAGCAGCCTTTCGT	[36]
*gbpD*-r	CTTCTGGTTGATAGGCGGCA	
*brpA*-f	GGAGGAGCTGCATCAGGATTC	[33]
*brpA*-r	AACTCCAGCACATCCAGCAAG	
*luxS*-f	ACTGTTCCCCTTTTGGCTGTC	[35]
*luxS*-r	AACTTGCTTTGATGACTGTGGC	
*comDE*-f	ACAATTCCTTGAGTTCCATCCAAG	[37]
*comDE*-r	TGGTCTGCTGCCTGTTGC	
*comX*-f	CGTCAGCAAGAAAGTCAGAAAC	[38]
*comX*-r	ATACCGCCACTTGACAAACAG	
*vicR*-f	CGTGTAAAAGCGCATCTTCG	[34]
*vicR*-r	AATGTTCACGCGTCATCACC	

## Data Availability

The datasets generated and analyzed to support the study are available from the corresponding author on reasonable request.
